# Elevated matrix metalloproteinase‐9 levels in neuronal extracellular vesicles in Alzheimer’s disease

**DOI:** 10.1002/acn3.51155

**Published:** 2020-08-13

**Authors:** Dongmei Gu, Fang Liu, Meng Meng, Liling Zhang, Marc L. Gordon, Ying Wang, Li Cai, Nan Zhang

**Affiliations:** ^1^ Department of Clinical Laboratory Tianjin Medical University General Hospital Tianjin China; ^2^ Department of Clinical Laboratory Tianjin Medical University General Hospital Airport Hospital Tianjin China; ^3^ Department of Neurology Tianjin Medical University General Hospital Tianjin China; ^4^ Department of Neurology Tianjin Medical University General Hospital Airport Hospital Tianjin China; ^5^ Department of Neurology Heji Hospital Affiliated to Changzhi Medical College Changzhi Shanxi China; ^6^ The Litwin‐Zucker Research Center The Feinstein Institutes for Medical Research Northwell Health 350 Community Drive Manhasset New York; ^7^ Donald and Barbara Zucker School of Medicine at Hofstra/Northwell 500 Hofstra Blvd Hempstead New York; ^8^ Department of PET‐CT Diagnostic Tianjin Medical University General Hospital Tianjin China

## Abstract

**Objective:**

This study aimed to investigate plasma neuronally derived extracellular vesicle (NDEV) levels of core pathological markers [amyloid‐β (Aβ) and phosphorylated tau] and inflammatory biomarkers, including interleukin 6 (IL‐6) and matrix metalloproteinase‐9 (MMP‐9) in patients with Alzheimer’s disease (AD).

**Methods:**

Thirty‐one patients with AD and 15 cognitively normal controls (NCs) were recruited. The diagnosis of AD was supported by fluorodeoxyglucose and Pittsburgh Compound‐B PET scans. Plasma extracellular vesicles were extracted, precipitated, and enriched for neuronal source by anti‐L1CAM antibody absorption. Levels of Aβ42, P‐T181‐tau, P‐S396‐tau, IL‐6, and MMP‐9 in plasma NDEVs were quantified by enzyme‐linked immunosorbent assay (ELISA).

**Results:**

Aβ42, P‐T181‐tau, and MMP‐9 levels in plasma NDEVs were significantly higher in patients with AD than NCs. However, P‐S396‐tau and IL‐6 levels in plasma NDEVs did not differ between AD patients and NCs. Moreover, there was no correlation between any of these biomarker levels and cognitive function as measured with Mini‐Mental State Examination in patients with AD.

**Conclusions:**

These findings provide further support that levels of core pathological markers, including Aβ42 and P‐T181‐tau, are elevated in plasma NDEVs of patients with AD. Furthermore, MMP‐9 might play an important role in the pathogenesis of AD, and is a promising inflammatory biomarker for AD.

## Introduction

Alzheimer’s disease (AD) is the most common cause of age‐related neurodegenerative disease, affecting millions of individuals worldwide. To date, assays of core neuropathology, for example, amyloid‐β (Aβ) and tau protein with positron emission tomography (PET) or in cerebrospinal fluid (CSF), have been validated to have a high accuracy for AD diagnosis in vivo, and have been included in recent criteria for AD research.^1^, ^2^ Even so, exploration of peripheral biomarkers is still imperative because PET scan is not available in most medical settings and the invasiveness of lumbar puncture limits its clinical application in patients with cognitive impairment.

Extracellular vesicles (EVs) are nanosized particles released from virtually all types of cells, including neurons, that circulate in the interstitial space, both in the brain and the periphery, and easily diffuse into biological fluids such as blood, urine, and CSF.^3^, ^4^ Because EVs contain proteins, messenger RNAs, and microRNAs from parent cells, they play a prominent role in cellular signaling, removal of unwanted proteins, and transfer of cellular pathogens.^5^ It has been reported that neuronally derived EVs (NDEVs), exosomes, in particular, contain amyloid precursor protein and its metabolites, for example, C‐terminal fragments, amyloid intracellular domain, and Aβ.^6^, ^7^ Tau aggregates in the brain could also be packaged inside exosomes, and then transported into CSF and even blood.^8^ The discovery of disease‐related proteins in EVs isolated from AD patient plasma/CSF samples has stimulated research aiming to utilize EVs as biomarkers.^9^

Elevated levels of Aβ42 and phosphorylated tau (P‐T181‐tau and P‐S396‐tau) in plasma NDEVs were observed in patients with AD even before symptom onset, and could predict AD conversion from mild cognitive impairment (MCI).^10^, ^11^ Recently, it was demonstrated that Aβ42, total tau (T‐tau), and P‐T181‐tau levels were highly correlated between plasma NDEVs and CSF in a multicenter study.^12^ Moreover, Goetzl and colleagues found that plasma NDEV levels of several proteins, such as phosphorylated type 1 insulin receptor substrate (P‐S312‐IRS‐1 and P‐panY‐IRS‐1),^13^ lysosomal proteins (Cathepsin D, lysosome‐associated membrane protein 1, ubiquitin, and heat‐shock protein 70),^14^ cellular survival factors (low‐density lipoprotein receptor‐related protein 6, heat‐shock factor‐1, and repressor element 1‐silencing transcription factor),^15^ and synaptic proteins (synaptophysin, synaptopodin, synaptotagmins, neurogranin, and growth‐associated protein 43),^16^ were different between patients with AD and cognitively normal controls (NCs) in preliminary studies. However, although there is extensive evidence that neuroinflammation plays a significant role in the pathogenesis of AD,^17^ biomarkers of inflammation have not previously been investigated by analyzing plasma NDEVs.

In this study, we extracted plasma NDEVs from patients with fluorodeoxyglucose (FDG) and amyloid PET supported diagnoses of AD and NCs. We aimed to investigate plasma NDEV levels of core pathological markers (Aβ42, P‐T181‐tau, and P‐S396‐tau) and inflammatory biomarkers, including interleukin 6 (IL‐6) and matrix metalloproteinase‐9 (MMP‐9) in AD patients.

## Methods

### Study design and participants

This study was approved by the Ethics Committee of Tianjin Medical University General Hospital. Written informed consent was obtained from all participants. Plasma samples were acquired from 46 subjects, including 31 patients with AD (mean age, 68.58 ± 8.04) and 15 cognitively NCs (mean age, 64.80 ± 6.00) recruited from Tianjin Medical University General Hospital. All participants had apolipoprotein E (APOE) genotype testing, except for three AD patients who were not able to return for this test.

The inclusion criteria for AD group were as follows: (1) all patients met the research diagnostic criteria of International Working Group‐2 (IWG‐2) for typical AD.^1^ Briefly, all AD patients had significant episodic memory impairment and an amyloid‐positive ^11^C‐labeled Pittsburgh Compound‐B (PiB) PET scan; (2) age range from 50 to 85; (3) Clinical Dementia Rating (CDR) score = 0.5–2, Mini‐Mental State Examination (MMSE) score less than 27. The exclusion criteria included (1) cognitive impairment caused by other neurological diseases or mental disorders, such as cerebrovascular disease, Parkinson’s disease, or dementia with Lewy bodies, frontotemporal lobar degeneration (FTLD), multiple sclerosis, traumatic brain injury, brain tumor, or schizophrenia; (2) moderate to severe depressed mood (score > 16 on the Chinese version of the Beck Depression Inventory‐II); and (3) other conditions affecting cognitive function, for example, abnormal thyroid function or vitamin B 12 deficiency. Age‐ and gender‐matched NCs had no complaint of memory or cognitive decline, and a CDR score of 0 and an MMSE score of more than 24. In addition, there was no clinically significant brain atrophy or evidence of cerebrovascular disease on magnetic resonance imaging (MRI) in NCs.

**Figure 1:**
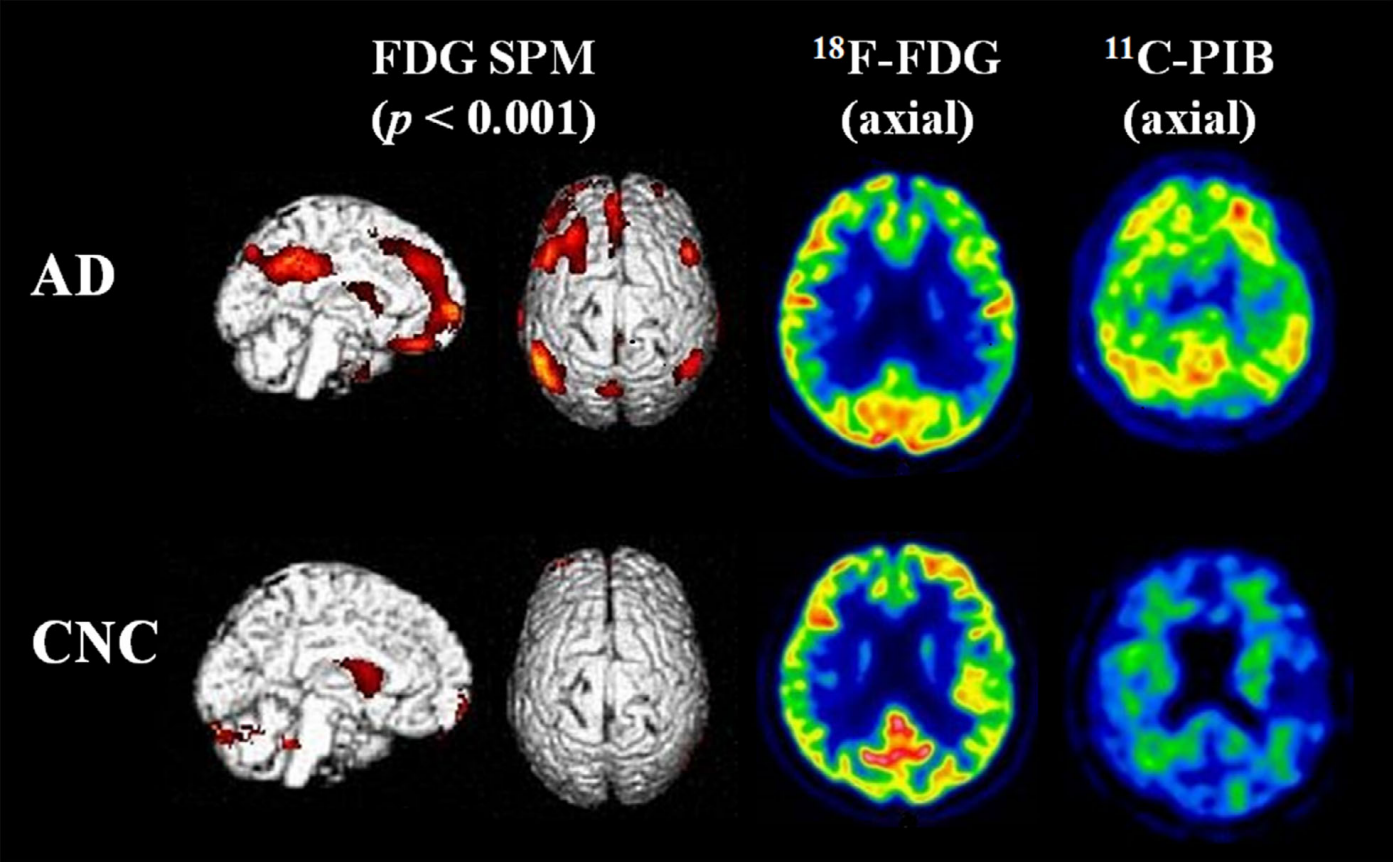
Representative FDG and PiB PET patterns in patients with AD and NCs. The left two rows show SPM T‐maps (*P* < 0.001) rendered on the ch2bet template brain. The right two rows display axial images of FDG PET and PiB PET, respectively. Hypometabolism in inferior parietal lobule, precuneus/posterior cingulate gyrus, bilateral dorsolateral frontal lobe, and medial prefrontal cortex on FDG PET and amyloid positivity on PiB PET are seen in a patient with AD. Normal glucose metabolism on FDG PET and amyloid negativity on PiB PET from a healthy individual are also presented. Abbreviations: FDG, fluorodeoxyglucose; SPM, statistical parametric mapping; PiB, Pittsburgh Compound‐B; PET, positron emission tomography; AD, Alzheimer’s disease; NC, normal control.

FDG PET and PiB PET scans were performed at the PET/CT center of Tianjin Medical University General Hospital on a GE 710 PET/CT scanner in the three‐dimensional scanning mode. The procedure for PET imaging acquisition and analysis was completed according to our previous publications.^18^, ^19^ Briefly, patients were fasted for at least 6 hours before tracer injection with plasma glucose level being within the normal range. FDG PET was conducted 1 hour after PiB PET scan at the same PET facility. Target‐to‐cerebellum ratios of ^11^C‐PiB uptake were calculated for 11 bilateral regions. PiB PET images were judged either positive or negative based on the reference value of standardized uptake value ratio from our previous analysis of healthy individuals. FDG frames for each subject were summed and normalized to mean activity in the pons, then were presented in the NIH color scale, and could be windowed and viewed in three planes at the rater’s discretion. FDG PET images were rated as “AD” if hypometabolism was mostly seen in temporoparietal cortex. All images were rated by two experienced nuclear medicine physicians who were blind to the clinical data. Representative images for FDG PET and PiB PET of patients with AD from this study are shown in Figure 1.

### NDEVs isolation from plasma

The isolation and assays of NDEVs were performed according to previously described methods by Goetzl et al.^10^ Specifically, 3 mL of venous blood was drawn into a 5 mL tube of saline with ethylenediaminetetraacetic acid (EDTA) and incubated for 10 minutes at room temperature. The whole blood was centrifuged at 2500 *g* for 15 minutes at 4℃. The plasma was transferred to storage tubes (Solarbio, Science & Technology Co.Ltd. BJ) as 500 µL aliquots and frozen immediately in a −80℃ freezer.

One‐half milliliter of plasma was incubated with 0.15 mL of thromboplastin‐D (Fisher Scientific, Inc., HanoverPark, IL) at room temperature for 60 minutes, followed by the addition of 0.35 mL of calcium‐ and magnesium‐free Dulbecco’s balanced salt solution (DBS^‐2^) with protease inhibitor cocktail (Roche Applied Sciences, Inc., Indianapolis, IN) and phosphatase inhibitor cocktail (Pierce Halt, Thermo Scientific, Inc., Rockford, IL). After centrifugation at 3000× *g* for 20 minutes, supernates were mixed with 252 µL of ExoQuick exosome precipitation solution (EXOQ; System Biosciences, Inc., Mountain view, CA), and incubated for 1 hour at 4℃. The resultant EV suspensions were centrifuged at 1500× *g* for 30 minutes at 4℃ and each pellet was resuspended in 350 µL of DBS^‐2^ with inhibitor cocktails.

Each sample was mixed with 50 µL of 3% bovine serum albumin (Thermo Scientific, Inc.) and was incubated for 1 hour at 4℃ with 1 µg of mouse anti‐human CD171 antibody (L1CAM neural adhesion protein) (eBio5G3, eBioscience, San Diego, CA); then was followed by addition of 25 μL streptavidin‐agarose resin (Thermo Scientific, Inc.) plus 50 μL of 3% BSA for 30 minutes at 4°C. After centrifugation at 400 *g* for 10 minutes at 4°C and removal of the supernates, each pellet was suspended in 50 μL of 0.05 M glycine‐HCl (pH 3.0) by vortex mixing for 10 seconds. Each suspension was then combined with 0.5 mL M‐PER mammalian protein extraction reagent that had been adjusted to pH 8.0 with 1 M Tris‐HCl (pH 8.6) and the inhibitor cocktails followed by incubation for 10 minutes at 37°C with vortex mixing for 15 seconds. And then the samples were stored at −80℃ before enzyme‐linked immunosorbent assay (ELISA).

### Characterization of plasma NDEVs based on shape and size

L1CAM‐positive plasma NDEVs were characterized according to their sizes and shapes by transmission electron microscopy (TEM), using a Talos F200c electron microscope (FEI, USA) at an acceleration voltage of 200 kV. In addition, NDEVs were pooled in 1mL DBS^‐2^, diluted 1:200, and visualized with a NanoSight 500 instrument (Nano Sight, Amesbury, UK) and characterized with nanoparticle tracking analysis (NTA) version 2.3 based on size distribution.

### Characterization of plasma NDEVs by protein composition measured with Western blotting

The NDEV supernatant was denatured in 5× sodium dodecyl sulfonate (SDS) buffer and subjected to Western blotting analysis (10% SDS‐polyacrylamide gel electrophoresis; 50 µg protein/lane) using rabbit polyclonal antibody for ALIX (sc‐53540, Santa Cruz, CA, USA), HSP90 (60318‐I‐Ig, Proteintech, Rosemont, IL), CD63 (sc‐5275, Santa Cruz, CA, USA), and calnexin (10427‐2‐AP, Promega, Madison, WI). The proteins were visualized on the Tanon4600 Automatic chemiluminescence image analysis system (Tanon, Shanghai, China).

### ELISA quantification of NDEV proteins/peptides

NDEV proteins/peptides were quantified by ELISA kits for human Aβ42 (Life Technologies/Invitrogen, Camarillo, CA), human P‐T181‐tau (Innogenetics Division of Fujire bio US, Inc., Alpharetta, GA), human P‐S396‐tau (Life Technologies/Invitrogen, Camarillo, CA), human IL‐6 (Merck Millipore, MA), and human MMP‐9 (Life Technologies/Invitrogen, Camarillo, CA) according to suppliers’ directions. CD81, which is a canonical protein marker for EVs,^20^ was also measured by ELISA (Cusabio‐American Research Products, Inc.) to normalize the quantification of NDEV proteins/peptides. The mean value for all determinations of CD81 in each assay group was set at 1.00 and the relative values for each sample used to normalize their recovery.

### Statistical analyses

The differences in demographics and protein/peptide concentrations in plasma NDEVs between AD patients and NCs were determined with chi‐square tests for categorical variables or independent two samples t‐test for continuous variables. We also tested for age and group (AD and NC) interaction for each NDEV protein/peptide with general linear model to assess the potential effect of age. Sensitivity and specificity of plasma NDEV proteins/peptides with significant differences between the two groups were evaluated using receiver operating characteristic (ROC) analyses to determine their performances for discriminating patients with AD and NCs. The correlations between NDEV proteins/peptides and MMSE score were analyzed using Pearson correlation analysis. GraphPad Prism 6 was used for scatter plots, and SPSS v21.0 was used for data analysis. Values of *P* < 0.05 were regarded as statistically significant.

## Results

### Demographics of all participants

The demographics and clinical characteristics of patients with AD and NCs are shown in Table 1. There was no significant difference in age, gender, and level of education between patients with AD and NCs. The AD group had a significantly lower MMSE score than the NC group (15.93 ± 6.61 vs 27.67 ± 1.72, *P* < 0.001). The proportion of APOE *ε*4 allele carriers, whether homozygous or heterozygous with an *ε*3 allele, was much higher in the AD group (42.86%, data missing for three patients) than the NC group (13.33%) (*P* < 0.05).

**Table 1:** Demographics and clinical characteristics of patients with AD and NCs.

	AD N = 31	NC N = 15	*χ^2^*/*t*	*P*
Gender, male/female	8/23	5/10	0.282	0.730
Age, y	68.58 ± 8.04	64.80 ± 6.00	1.613	0.114
Education, y	10.06 ± 4.49	12.60 ± 2.35	‐1.740	0.089
MMSE	15.93 ± 6.61	27.67 ± 1.72	‐6.724	0.000
APOE ε4 carrier (%)	12/28 (42.86)	2/15 (13.33)	4.710	0.045

Age, education, and MMSE score are provided as mean ± SD and analyzed using independent two sample t‐test. Chi‐square test was used for analysis of gender and APOE genotype. APOE genotype data were missing from three AD patients. Abbreviations: AD, Alzheimer’s disease; NC, normal control; MMSE, Mini‐Mental State Examination; APOE, apolipoprotein E.

### Characterization of L1CAM‐positive plasma NDEVs

**Figure 2:**
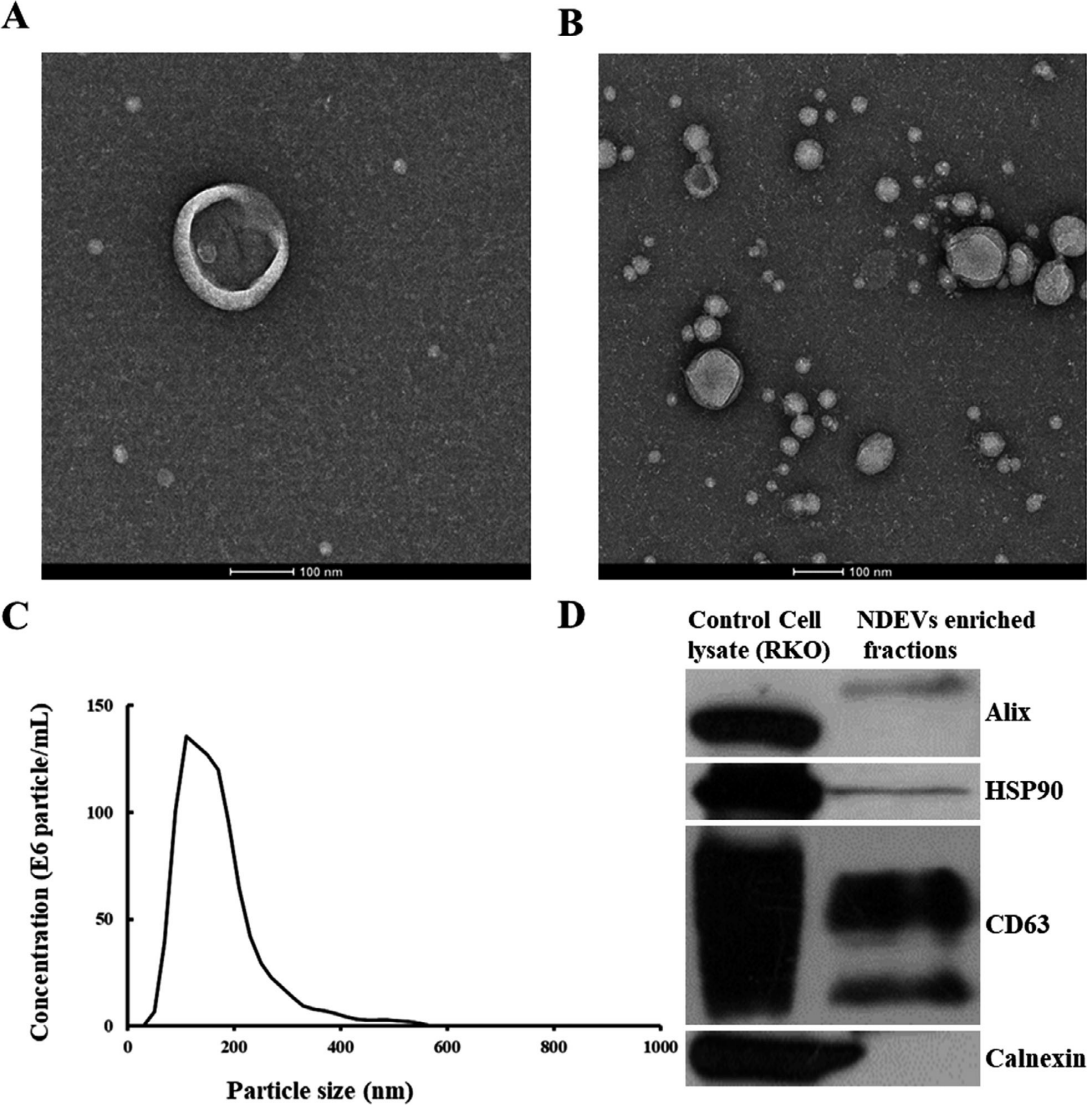
Plasma NDEVs identified with TEM, NTA, and Western blotting. A single‐plasma NDEV (A) and clusters of plasma NDEVs (B) were measured with TEM. Scale bars equal 100 nm. An NTA plot (C) of size/concentration for plasma NDEVs derived from a patient with AD shows a high concentration. (D) ALIX, HSP90, and CD63 (EV markers) were present and calnexin (a marker for cellular components) was absent in plasma NDEVs enriched fraction samples. Abbreviations: NDEV, neuronally derived extracellular vesicle; TEM, transmission electron microscopy; NTA, nanoparticle tracking analysis; AD, Alzheimer’s disease.

Plasma NDEVs were identified according to morphology and size distribution using TEM and NTA system, respectively, and presence of specific EV markers and absence of cellular components measured with Western blotting. TEM showed that plasma NDEVs varied in size and usually had a diameter of less than 150 nm (Fig. 2A,B). NTA indicated a high concentration of plasma NDEVs, which was up to 191.150 particles/mL (Fig. 2C). Enrichment of EV markers ALIX, HSP90 and CD63 was all detected, and calnexin, a negative marker of EVs, was absent in plasma NDEVs enriched fraction samples (Fig. 2D).

### Comparison of NDEV protein levels between patients with AD and NCs

**Figure 3:**
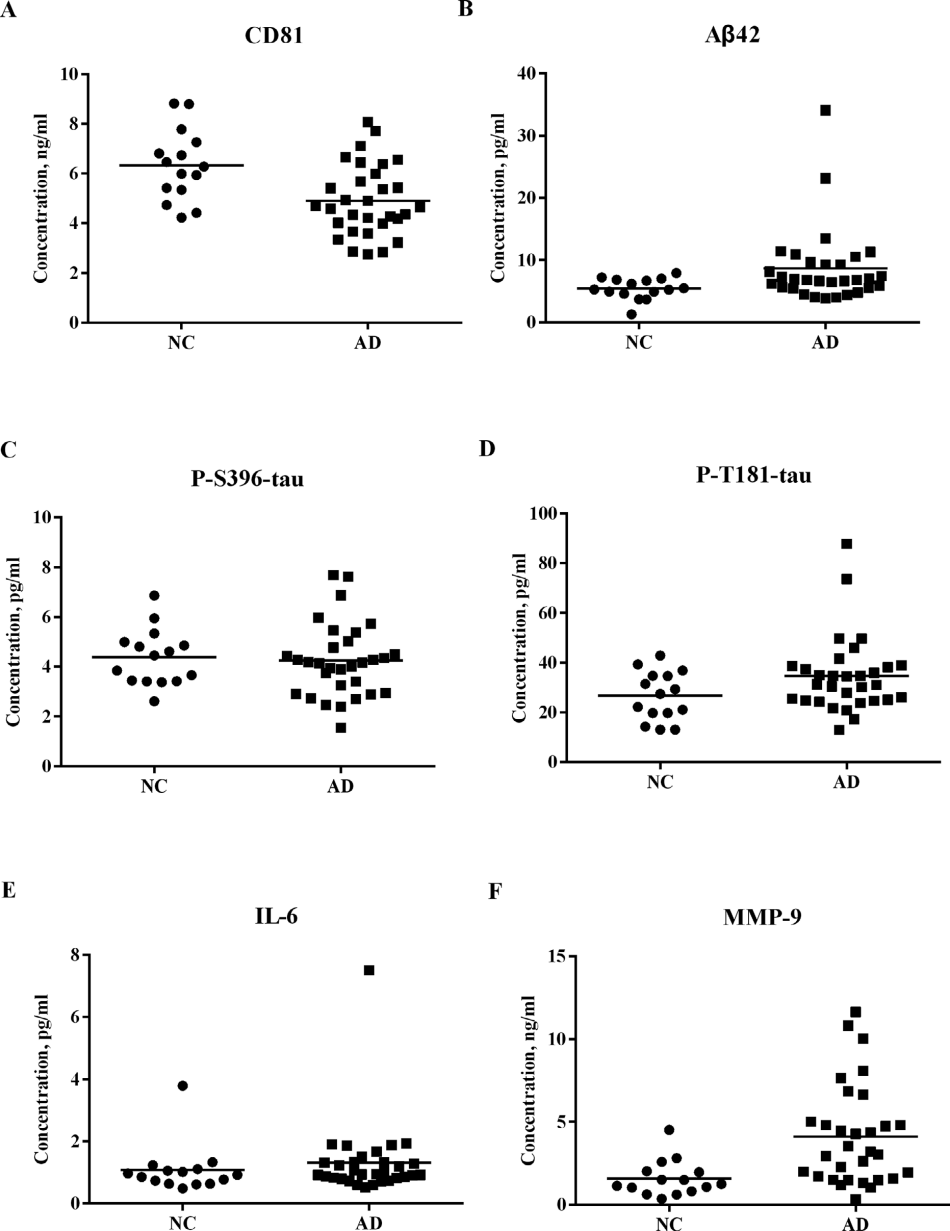
NDEV protein levels in patients with AD and NCs. CD81 levels in plasma NDEVs (A) were lower in patients with AD than NCs (*P* < 0.05). CD81 normalized levels of Aβ42 (B), P‐T181‐tau (D), and MMP‐9 (F) levels in plasma NDEVs were significantly higher in patients with AD than NCs (*P* < 0.05). There was no difference in P‐S396‐tau (C) and IL‐6 (E) levels between patients with AD and NCs. Abbreviations: NDEV, neuronally derived extracellular vesicle; AD, Alzheimer’s disease; NC, normal control; IL‐6, interleukin 6; MMP‐9, matrix metalloproteinase 9.

Levels of all NDEV proteins from AD patients and NCs are shown in Table 2. The AD group had decreased levels of the EV membrane marker protein CD81 compared to NCs (*P* < 0.05) (Fig. 3A). CD81 normalized levels of Aβ42 (8.64 ± 6.037 pg/mL vs 5.40 ± 1.700 pg/mL, *P* < 0.05), P‐T181‐tau (34.68 ± 15.198 pg/mL vs 26.68 ± 9.816 pg/mL, *P* < 0.05), and MMP‐9 (4.11 ± 3.006 ng/mL vs 1.60 ± 1.078 ng/mL, *P* < 0.01) in plasma NDEVs were elevated in patients with AD as compared with NCs (Fig. 3B,D,F). There was no difference in P‐S396‐tau and IL‐6 levels in plasma NDEVs between the AD group and the NC group (Fig. 3C,E). Moreover, there was no significant interaction effect of age and group on any NDEV protein/peptide, suggesting that the results did not differ by age.

**Table 2:** Levels of NDEV proteins/peptides in patients with AD and NCs.

	AD N = 31	NC N = 15	*t*	*P*
CD81 (ng/mL)	4.91 ± 1.426	6.27 ± 1.580	−2.917	0.006
Aβ42 (pg/mL)	8.64 ± 6.037	5.40 ± 1.700	2.027	0.049
P‐S396‐tau (pg/mL)	4.25 ± 1.474	4.38 ± 1.135	−0.299	0.766
P‐T181‐tau (pg/mL)	34.68 ± 15.198	26.68 ± 9.816	2.049	0.047
IL‐6 (pg/mL)	1.32 ± 1.220	1.08 ± 0.787	0.683	0.498
MMP‐9 (ng/mL)	4.11 ± 3.006	1.60 ± 1.078	4.146	<0.001

Values are provided as mean ± SD. The differences in NDEV protein/peptide levels between patients with AD and NCs were determined by independent two sample t‐test. Abbreviations: NDEV, neuronally derived extracellular vesicle; AD, Alzheimer’s disease; NC, normal control; IL‐6, interleukin 6; MMP‐9, matrix metalloproteinase‐9.

### ROC analysis of NDEV proteins/peptides for discrimination between AD patients and NCs

**Figure 4:**
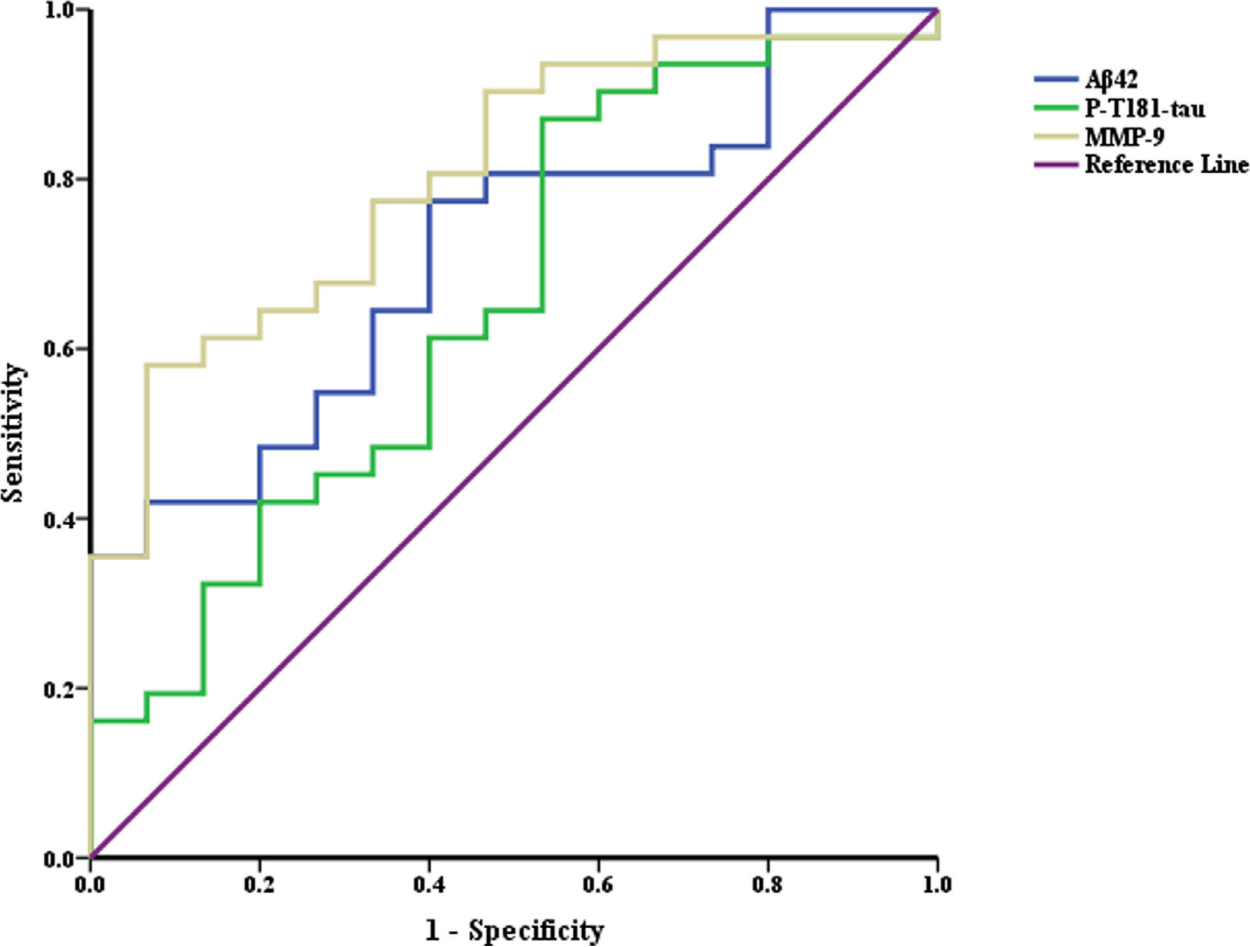
ROC curves of Aβ42, P‐T181‐tau, and MMP‐9 in plasma NDEVs for discrimination between patients with AD and NCs. The AUC values for Aβ42, P‐T181‐tau, and MMP‐9 were 0.71, 0.66, and 0.80, respectively. The sensitivity and specificity were 77.42% (95% CI 58.46‐89.72) and 60.00% (95% CI 32.89‐82.54) for Aβ42 at a cutoff of 5.51 pg/mL, 80.65% (95% CI 61.94‐91.88) and 55.33% (95% CI 27.42‐77.72) for P‐T181‐tau at a cutoff of 22.96 pg/mL, and 61.29% (95% CI 42.29‐77.58) and 93.33% (95% CI 66.03‐99.65) for MMP‐9 at a cutoff of 2.88 ng/mL. Abbreviations: ROC, receiver operating characteristic; MMP‐9, matrix metalloproteinase 9; NDEVs, neuronally derived extracellular vesicles; AD, Alzheimer’s disease; NCs, normal controls; AUC, area under the curve.

ROC analyses of Aβ42, P‐T181‐tau, and MMP‐9, which significantly differed between patients with AD and NCs, were conducted to determine the sensitivity and specificity for AD diagnosis (Fig. 4). The area under the curve (AUC) values for Aβ42, P‐T181‐tau, and MMP‐9 were 0.71, 0.66, and 0.80 at a cutoff of 5.51 pg/mL, 22.96 pg/Ml, and 2.88 ng/mL, respectively. The sensitivity, specificity, positive predictive value, and negative predictive value for Aβ42 were 77.42% (95% CI 58.46‐89.72), 60.00% (95% CI 32.89‐82.54), 80.00% (95% CI 60.87‐91.60), and 56.25% (95% CI 30.55‐79.25), respectively; for P‐T181‐tau were 80.65% (95% CI 61.94‐91.88), 55.33% (95% CI 27.42‐77.72), 78.13% (95% CI 59.56‐90.06), and 57.14% (95% CI 29.65‐81.19), respectively; and for MMP‐9 were 61.29% (95% CI 42.29‐77.58), 93.33% (95% CI 66.03‐99.65), 95.00% (95% CI 73.06‐99.74), and 53.85% (95% CI 33.75‐72.86), respectively.

### Association between NDEV protein levels and severity of cognitive impairment in patients with AD

There was no correlation between any protein/peptide level in plasma NDEVs, including Aβ42, P‐T181‐tau, P‐S396‐tau, IL‐6, and MMP‐9, and cognitive function measured with MMSE in patients with AD (Table 3).

**Table 3:** Correlation between NDEV proteins/peptides and MMSE score in patients with AD.

	Aβ42 (pg/mL)	P‐S396‐tau (pg/mL)	P‐T181‐tau (pg/mL)	IL‐6 (pg/mL)	MMP‐9 (ng/mL)
*r*	0.017	0.089	0.271	−0.103	−0.232
*P*	0.929	0.641	0.147	0.589	0.217

Abbreviations: AD, Alzheimer’s disease;IL‐6, interleukin 6; MMP‐9, matrix metalloproteinase 9; MMSE, Mini‐Mental State Examination; NDEV, neuronally derived extracellular vesicle.

## Discussion

To our knowledge, this is the first study to evaluate Aβ, tau, and biomarkers of inflammation in plasma NDEVs in patients with amyloid PET supported AD. We observed that levels of Aβ42, P‐T181‐tau, and MMP‐9 in plasma NDEVs were elevated in patients with AD as compared with NCs. Moreover, at their optimal cutoff value, Aβ42 and P‐T181‐tau showed a high sensitivity of 77.42% and 80.65%, and a moderate specificity of 60.00% and 55.33%, respectively; on the contrary, MMP‐9 had a high specificity of 93.33% and a moderate sensitivity of 61.29%. However, there was no correlation between MMSE score and any core pathological biomarker or inflammatory biomarker in patients with AD.

As neuropathological hallmarks of AD, alterations of Aβ42 and phosphorylated tau in the CSF have been consistently demonstrated and are included in diagnostic criteria for AD research.^2^ EV‐mediated secretion may play a significant role in the abnormal processing and transport of Aβ and tau in the brain, especially at the early stage of AD.^21^, ^22^ P‐T181‐tau, the phosphorylated epitope that is most enriched in secreted (exosomal) tau, is an established biomarker for the elevated tau seen in early AD and is used in CSF‐based diagnostics for AD.^23^ Elevated levels of Aβ42 and P‐T181‐tau in plasma NDEVs have been reported in previous studies.^10^, ^11^ In a recent large cohort study, P‐T181‐tau and P‐T231‐tau in plasma NDEVs, quantified using an electrochemiluminescence assay, were able to differentiate participants with future AD from controls, whereas Aβ42 examined with SIMOA assay showed no difference between future AD participants and controls.^24^ We found that both Aβ42 and P‐T181‐tau levels in plasma NDEVs measured with ELISA were elevated in patients with AD. However, P‐S396‐tau levels did not differ between AD patients and NCs in the present study, in contrast to previous studies.^10^, ^11^ Phosphorylation of tau at S396 shows higher variability than other p‐tau sites, and starts very late and to a limited degree in the Braak stage progression of AD.^25^

MMPs, which are calcium‐ and zinc‐dependent endopeptidases of the met zincin superfamily, participate in many physiological and pathological processes in the central nervous system.^26^ Post mortem studies observed increased MMP‐9 expression in different brain tissues of patients with AD, such as the cytoplasm of neurons, neurofibrillary tangles, amyloid plaques, and vascular walls of hippocampal and cerebral cortex.^27^ However, previous findings on MMP‐9 levels in the plasma or CSF of AD patients have been inconsistent. A recent proteomics study observed elevated plasma MMP‐9 levels in patients with MCI compared to Aβ negative cognitively NCs.^28^ On the contrary, normal or decreased MMP‐9 level in the CSF was observed in patients with AD or MCI in other studies.^29^, ^30^ In the present study, we observed that MMP‐9 level in plasma NDEVs was significantly higher in patients with AD than NCs, indicating that MMP‐9 is involved in AD progression and could potentially be a biomarker for AD. Regarding the pathogenesis of AD, several studies reported that MMP‐9 has either beneficial or detrimental effects on a variety of pathophysiological processes, such as mediating Aβ degradation and stimulating astrocyte activation, increasing brain‐derived neurotrophic factor level and maintaining synaptic plasticity, as well as regulating neuroinflammation.^31^

IL‐6 as a pro‐inflammatory cytokine, which may accelerate ongoing neurodegenerative processes in AD, has been extensively investigated in preclinical and clinical studies.^32^ Post mortem studies showed elevated IL‐6 levels in AD brain,^33^ and it may increase slowly over the course of the disease.^34^ However, a meta‐analysis indicated that IL‐6 levels were increased in the periphery, but not in CSF of patients with AD.^35^ Likewise, we did not find a difference in IL‐6 levels between AD patients and NCs by measuring plasma NDEVs, which are considered to reflect changes in the brain. Large interindividual variances of IL‐6 levels in previous studies, sometimes ranging from 50 to 100% of the reported mean values, could predispose to contradictory findings when comparing small patient cohorts.^32^

Post mortem studies have shown that both Aβ plaques and neurofibrillary tangles are correlated with ante mortem cognitive impairment in patients with AD.^36^ However, since these aggregates, especially Aβ, could be reabsorbed or removed, they might plateau during disease progression, leading to a “ceiling effect” at the early stage.^37^ Moreover, higher P‐T181‐tau level in plasma NDEVs was found to be associated with worse verbal memory, attention, executive function, and visuospatial function in a large cohort.^24^ Levels of inflammatory biomarkers, which may play either beneficial or detrimental roles in AD pathogenesis, may undergo a complicated dynamic evolution during disease progression. Previous studies on the association between inflammation and cognitive decline have been inconsistent.^38^ For instance, higher peripheral IL‐6 levels correlated with greater cognitive deficits in some studies.^38^, ^39^ However, no correlation was observed in other studies.^40^ We did not find any correlation between NDEV protein/peptide levels and severity of cognitive impairment measured with MMSE in patients with AD. Longitudinal studies may provide more reliable data clarifying how the proteins in plasma NDEVs change over time across different clinical and pathological stages of AD.

The concentration of NDEVs defined with CD81 was different between the AD group and the NC group in this study. There may be several reasons for this observation. First, the plasma samples from the AD group were stored for a much longer time (a few samples for more than 2 years) than the NCs. It was reported that urinary exosome concentrations decline with increasing duration of storage.^41^ Moreover, compared with risk‐neutral APOE *ε*3 allele homozygotes in humans or humanized mouse models, the *ε*4 allele, whether homozygous or heterozygous with an *ε*3 allele, caused decreased exosome levels in the extracellular space of the brain.^42^ In the present study, the AD group had a much higher proportion of APOE *ε*4 allele carriers than the NC group.

Our findings from amyloid PET supported AD patients add important information to the existing literature on NDEV‐based biomarkers of AD, but some limitations need to be noted regarding the study design and methodology. First, the levels of some proteins were relatively low, especially P‐S396‐tau and IL‐6. More sensitive methods for protein measurement, such as ultrasensitive SIMOA immunoassay and chromatography‐mass spectrometry assay, are suggested in future studies. For instance, NDEV biomarkers of phosphorylated tau and insulin receptor substrate 1 were validated with electrochemiluminescence and SIMOA assays in a large cohort as mentioned above.^24^ Second, proteins in NDEVs are possibly altered by drugs or conditions encountered in transition from the original parent cell to the ultimate target,^43^ which were not taken into account in this study. Third, although a comprehensive neuropsychological assessment and a brain MRI scan were used for screening NCs, pathological alterations of neurodegenerative diseases, such as AD, and cerebrovascular disorders could potentially exist in some cognitively healthy individuals. Fourth, cognitive function was only assessed by MMSE, which is a brief test for screening and evaluation purposes. Assessment of different cognitive domains was not analyzed in this study. Finally, since the sample size is small, our results need to be validated in larger cohorts.

In conclusion, levels of core pathological markers, such as Aβ42 and P‐T181‐tau, in plasma NDEVs were elevated in patients with amyloid PET supported AD. Furthermore, MMP‐9 in plasma NDEVs, as a marker of inflammation, might play an important role in the pathogenesis of AD and is a promising biomarker for AD identification.

## Conflict of Interest

Within the past 2 years, MG has received research support without direct compensation from MSD (Merck), Eisai, AbbVie, and Janssen. MG has also served on an advisory board for Eisai. The remaining authors declare that they have no competing interests.

## Author Contributions

NZ conceived and designed the study. DG, MM, and LZ performed the assays of neuronally derived extracellular vesicles and acquired the data. DG analyzed the data and wrote the manuscript. FL, MM, and NZ recruited and assessed the participants. YW and LC conducted the PET procedure and read the images. NZ and MG substantively revised the manuscript for important intellectual content. All authors read and approved the final manuscript.
